# 5-(4-Eth­oxy­phen­yl)-3-(pyridin-2-yl)-4,5-dihydro-1*H*-pyrazole-1-carbothio­amide

**DOI:** 10.1107/S1600536812006642

**Published:** 2012-02-24

**Authors:** Suchada Chantrapromma, Phonpawee Nonthason, Thitipone Suwunwong, Hoong-Kun Fun

**Affiliations:** a Crystal Materials Research Unit, Department of Chemistry, Faculty of Science, Prince of Songkla University, Hat-Yai, Songkhla 90112, Thailand; b X-ray Crystallography Unit, School of Physics, Universiti Sains Malaysia, 11800 USM, Penang, Malaysia

## Abstract

In the title compound, C_17_H_18_N_4_OS, a pyrazoline derivative, the pyrazoline ring adopts an envelope conformation with the C atom bonded to the benzene ring as the flap atom. The dihedral angle between the pyridine and benzene rings is 80.50 (6)°. The eth­oxy­phenyl group is approximately planar, with an r.m.s. deviation of 0.0238 (1) Å for the nine non-H atoms. In the crystal, mol­ecules are linked by N—H⋯O and N—H⋯S hydrogen bonds into a tape along the *b* axis. Weak C—H⋯N and C—H⋯π inter­actions are also observed.

## Related literature

For bond-length data, see: Allen *et al.* (1987 ▶). For related literature on ring conformations, see: Cremer & Pople (1975 ▶). For related structures, see: Fun *et al.* (2012 ▶); Nonthason *et al.* (2011 ▶). For background to and applications of pyrazoline derivatives, see: Amir *et al.* (2008 ▶); Gong *et al.* (2011 ▶); Husain *et al.* (2008 ▶); Manna & Agrawal (2009 ▶); Özdemir *et al.* (2007 ▶); Sarkar *et al.* (2010 ▶). For the stability of the temperature controller used in the data collection, see: Cosier & Glazer (1986 ▶).
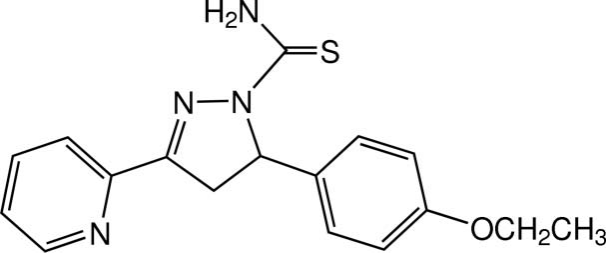


## Experimental

### 

#### Crystal data

C_17_H_18_N_4_OS
*M* *_r_* = 326.42
Monoclinic, 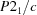

*a* = 13.4622 (1) Å
*b* = 9.4175 (1) Å
*c* = 13.3002 (2) Å
β = 103.146 (1)°
*V* = 1642.01 (3) Å^3^
*Z* = 4
Mo *K*α radiation
μ = 0.21 mm^−1^
*T* = 100 K
0.34 × 0.22 × 0.20 mm

#### Data collection

Bruker APEXII CCD area-detector diffractometer
Absorption correction: multi-scan (*SADABS*; Bruker, 2005 ▶) *T*_min_ = 0.934, *T*_max_ = 0.960
23465 measured reflections
4784 independent reflections
3970 reflections with *I* > 2σ(*I*)
*R*_int_ = 0.028

#### Refinement

*R*[*F* ^2^ > 2σ(*F* ^2^)] = 0.040
*wR*(*F* ^2^) = 0.097
*S* = 1.03
4784 reflections
217 parameters
H atoms treated by a mixture of independent and constrained refinement
Δρ_max_ = 0.45 e Å^−3^
Δρ_min_ = −0.26 e Å^−3^

### 

Data collection: *APEX2* (Bruker, 2005 ▶); cell refinement: *SAINT* (Bruker, 2005 ▶); data reduction: *SAINT*; program(s) used to solve structure: *SHELXTL* (Sheldrick, 2008 ▶); program(s) used to refine structure: *SHELXTL*; molecular graphics: *SHELXTL*; software used to prepare material for publication: *SHELXTL* and *PLATON* (Spek, 2009 ▶).

## Supplementary Material

Crystal structure: contains datablock(s) global, I. DOI: 10.1107/S1600536812006642/is5069sup1.cif

Structure factors: contains datablock(s) I. DOI: 10.1107/S1600536812006642/is5069Isup2.hkl

Supplementary material file. DOI: 10.1107/S1600536812006642/is5069Isup3.cml

Additional supplementary materials:  crystallographic information; 3D view; checkCIF report

## Figures and Tables

**Table 1:** Hydrogen-bond geometry (Å, °) *Cg*1 and *Cg*2 are the centroids of the C1–C5/N3 and C9–C14 rings, respectively.

*D*—H⋯*A*	*D*—H	H⋯*A*	*D*⋯*A*	*D*—H⋯*A*
N4—H1N4⋯S1^i^	0.925 (19)	2.472 (19)	3.3803 (12)	167.3 (15)
N4—H2N4⋯O1^ii^	0.825 (18)	2.296 (18)	3.0604 (15)	154.2 (17)
C3—H3*A*⋯N3^iii^	0.95	2.51	3.4053 (18)	156
C2—H2*A*⋯*Cg*2^iii^	0.95	2.67	3.4401 (14)	138
C7—H7*A*⋯*Cg*1^iv^	0.99	2.69	3.4841 (13)	138

Symmetry codes: (i) 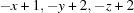; (ii) 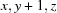; (iii) 
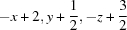
; (iv) 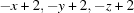.

**Fig. 1.**
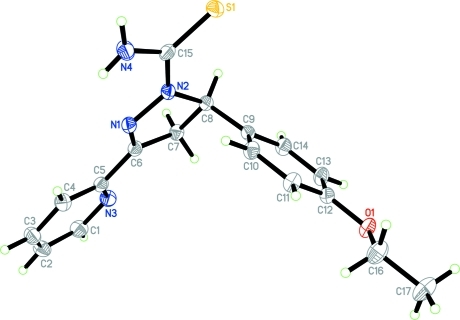
The molecular structure of the title compound, showing 60% probability displacement ellipsoids and the atom-numbering scheme.

**Fig. 2.**
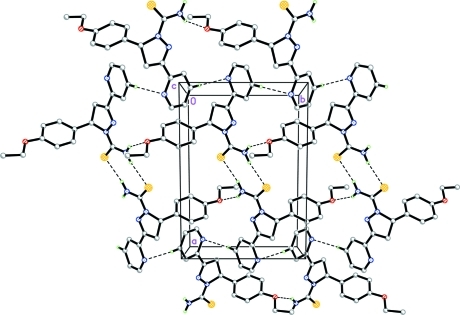
A packing diagram of the title compound viewed along the c axis. For the sake of clarity, only H atoms involved in the hydrogen bonds were shown. The hydrogen bonds were drawn as dashed lines.

## supplementary crystallographic information

### Comment

Pyrazoline derivatives are of interest in many fields, such as in medicinal chemistry due to their various bioactivities *i.e*. antidepressant and anticonvulsant (Özdemir *et al.*, 2007), antimicrobial (Manna & Agrawal, 2009), antiamoebic (Husain *et al.*, 2008), analgesic and anti-inflammatory (Amir *et al.*, 2008) properties, as well as being used as fluorescent sensors (Gong *et al.*, 2011) and fluorescent probes (Sarkar *et al.*, 2010). The title pyrazoline derivative (I) was synthesized because we want to modify the structure of heteroaryl chalcone derivative in order to enhance its fluorescence property, by cyclization with thiosemicarbazide. (I) possess fluorescent property as expected which will be reported elsewhere with its closely related compound (Nonthason *et al.*, 2011).

In the molecule of the title pyrazoline derivative (Fig. 1), C_17_H_18_N_4_OS, the pyrazoline ring adopts envelope conformation with the puckered C8 atom having the maximum deviation of 0.1408 (13) Å, and the puckering parameter Q = 0.2236 (13) Å and φ = 77.6 (3)° (Cremer & Pople, 1975). The dihedral angle between the pyridine and benzene ring is 80.50 (6)°. The conformation of the carbothioamide unit with respect to the pyrazoline ring can be indicated by the torsion angles N1–N2–C15–N4 = 1.90 (17)° and N1–N2–C15–S1 = -177.84 (8)°. The ethoxy group is co-planar with its attached benzene ring with the torsion angle C16–O1–C12–C11 = -0.39 (17)° and C12–O1–C16–C17 = 177.61 (11)°, and an r.m.s. deviation of 0.0238 (1) Å for the nine non H atoms (C9–C14/O1/C16/C17). Bond distances of (I) are in normal range (Allen *et al.*, 1987) and comparable with the related structures (Fun *et al.*, 2012; Nonthason *et al.*, 2011)

In the crystal packing (Fig. 2), the molecules are linked by N—H···O, and N—H···S hydrogen bonds into a tape along the *b* axis. Weak C—H···N and C—H···π interactions are also present (Table 1).

### Experimental

The title compound was synthesized by cyclization reaction of *E*-3-(4-ethoxyphenyl)-1-(pyridin-2-yl)prop-2-en-1-one (0.25 g, 1 mmol) with excess thiosemicarbazide (0.18 g, 2 mmol) in a solution of KOH (0.11 g, 2 mmol) in ethanol (10 ml). The reaction mixture was vigorously stirred and refluxed for 5 h. The pale-yellow solid of the title compound obtained after cooling of the reaction was then filtered off under vacuum. Pale yellow block-shaped single crystals of the title compound suitable for *X*-ray structure determination were recrystallized from ethanol by slow evaporation of the solvent at room temperature after several days (m.p. 480–481 K).

### Refinement

Amide H atoms were located in a difference maps and refined isotropically. The remaining H atoms were positioned geometrically and allowed to ride on their parent atoms, with C—H = 0.95 Å for aromatic, 1.00 Å for CH, 0.99 Å for CH_2_ and 0.98 Å for CH_3_ atoms. The *U*_iso_ values were constrained to be 1.5*U*_eq_ of the carrier atom for methyl H atoms and 1.2*U*_eq_ for the remaining H atoms. A rotating group model was used for the methyl groups.

### Crystal data

**Table:**

C_17_H_18_N_4_OS	*F*(000) = 688
*M**_r_* = 326.42	*D*_x_ = 1.320 Mg m^−^^3^
Monoclinic, *P*2_1_/*c*	Melting point = 480–481 K
Hall symbol: -P 2ybc	Mo *K*α radiation, λ = 0.71073 Å
*a* = 13.4622 (1) Å	Cell parameters from 4784 reflections
*b* = 9.4175 (1) Å	θ = 1.6–30.0°
*c* = 13.3002 (2) Å	µ = 0.21 mm^−^^1^
β = 103.146 (1)°	*T* = 100 K
*V* = 1642.01 (3) Å^3^	Block, pale yellow
*Z* = 4	0.34 × 0.22 × 0.20 mm

### Data collection

**Table:**

Bruker APEXII CCD area-detector diffractometer	4784 independent reflections
Radiation source: sealed tube	3970 reflections with *I* > 2σ(*I*)
Graphite monochromator	*R*_int_ = 0.028
φ and ω scans	θ_max_ = 30.0°, θ_min_ = 1.6°
Absorption correction: multi-scan (*SADABS*; Bruker, 2005)	*h* = −18→18
*T*_min_ = 0.934, *T*_max_ = 0.960	*k* = −13→11
23465 measured reflections	*l* = −18→13

### Refinement

**Table:**

Refinement on *F*^2^	Primary atom site location: structure-invariant direct methods
Least-squares matrix: full	Secondary atom site location: difference Fourier map
*R*[*F*^2^ > 2σ(*F*^2^)] = 0.040	Hydrogen site location: inferred from neighbouring sites
*wR*(*F*^2^) = 0.097	H atoms treated by a mixture of independent and constrained refinement
*S* = 1.03	*w* = 1/[σ^2^(*F*_o_^2^) + (0.0417*P*)^2^ + 0.7316*P*] where *P* = (*F*_o_^2^ + 2*F*_c_^2^)/3
4784 reflections	(Δ/σ)_max_ = 0.001
217 parameters	Δρ_max_ = 0.45 e Å^−^^3^
0 restraints	Δρ_min_ = −0.26 e Å^−^^3^

### Special details

**Table:**

Experimental. The crystal was placed in the cold stream of an Oxford Cryosystems Cobra open-flow nitrogen cryostat (Cosier & Glazer, 1986) operating at 120.0 (1) K.
Geometry. All esds (except the esd in the dihedral angle between two l.s. planes) are estimated using the full covariance matrix. The cell esds are taken into account individually in the estimation of esds in distances, angles and torsion angles; correlations between esds in cell parameters are only used when they are defined by crystal symmetry. An approximate (isotropic) treatment of cell esds is used for estimating esds involving l.s. planes.
Refinement. Refinement of F^2^ against ALL reflections. The weighted R-factor wR and goodness of fit S are based on F^2^, conventional R-factors R are based on F, with F set to zero for negative F^2^. The threshold expression of F^2^ > 2sigma(F^2^) is used only for calculating R-factors(gt) etc. and is not relevant to the choice of reflections for refinement. R-factors based on F^2^ are statistically about twice as large as those based on F, and R- factors based on ALL data will be even larger.

### Fractional atomic coordinates and isotropic or equivalent isotropic displacement parameters (Å^2^)

**Table:**

	*x*	*y*	*z*	*U*_iso_*/*U*_eq_	
S1	0.59440 (2)	0.80604 (3)	1.06339 (3)	0.01960 (9)	
O1	0.68026 (7)	0.21264 (9)	0.77613 (7)	0.01893 (19)	
N1	0.79666 (8)	0.91705 (11)	0.90593 (8)	0.0162 (2)	
N2	0.74574 (8)	0.84262 (11)	0.96929 (8)	0.0159 (2)	
N3	1.05784 (8)	0.87566 (12)	0.89934 (8)	0.0186 (2)	
N4	0.61973 (9)	1.00933 (12)	0.93150 (9)	0.0214 (2)	
H1N4	0.5594 (14)	1.0488 (19)	0.9404 (13)	0.033 (5)*	
H2N4	0.6522 (13)	1.0458 (19)	0.8925 (14)	0.032 (5)*	
C1	1.12851 (10)	0.92690 (15)	0.85186 (10)	0.0216 (3)	
H1A	1.1969	0.8950	0.8749	0.026*	
C2	1.10730 (10)	1.02328 (15)	0.77156 (10)	0.0228 (3)	
H2A	1.1596	1.0559	0.7399	0.027*	
C3	1.00762 (10)	1.07148 (15)	0.73821 (10)	0.0211 (3)	
H3A	0.9908	1.1381	0.6833	0.025*	
C4	0.93292 (9)	1.02150 (14)	0.78583 (10)	0.0187 (2)	
H4A	0.8643	1.0528	0.7644	0.022*	
C5	0.96155 (9)	0.92381 (13)	0.86619 (9)	0.0159 (2)	
C6	0.88757 (9)	0.86558 (13)	0.92109 (9)	0.0153 (2)	
C7	0.90988 (9)	0.75117 (13)	1.00217 (9)	0.0162 (2)	
H7A	0.9520	0.7875	1.0681	0.019*	
H7B	0.9445	0.6689	0.9789	0.019*	
C8	0.80072 (9)	0.71273 (13)	1.01217 (9)	0.0147 (2)	
H8A	0.7983	0.6995	1.0862	0.018*	
C9	0.76061 (9)	0.58201 (13)	0.94880 (9)	0.0142 (2)	
C10	0.68335 (9)	0.58700 (13)	0.85977 (9)	0.0167 (2)	
H10A	0.6505	0.6749	0.8388	0.020*	
C11	0.65281 (9)	0.46572 (13)	0.80034 (10)	0.0179 (2)	
H11A	0.5992	0.4710	0.7401	0.022*	
C12	0.70147 (9)	0.33741 (13)	0.83010 (9)	0.0159 (2)	
C13	0.77894 (10)	0.32974 (13)	0.91996 (10)	0.0184 (2)	
H13A	0.8118	0.2418	0.9409	0.022*	
C14	0.80746 (9)	0.45060 (13)	0.97800 (9)	0.0180 (2)	
H14A	0.8600	0.4447	1.0391	0.022*	
C15	0.65479 (9)	0.89184 (13)	0.98368 (9)	0.0159 (2)	
C16	0.60157 (10)	0.21507 (15)	0.68243 (11)	0.0246 (3)	
H16A	0.6181	0.2859	0.6337	0.029*	
H16B	0.5352	0.2406	0.6977	0.029*	
C17	0.59640 (12)	0.06844 (16)	0.63618 (12)	0.0306 (3)	
H17A	0.5425	0.0655	0.5728	0.046*	
H17B	0.5814	−0.0008	0.6857	0.046*	
H17C	0.6620	0.0453	0.6200	0.046*	

### Atomic displacement parameters (Å^2^)

**Table:**

	*U*^11^	*U*^22^	*U*^33^	*U*^12^	*U*^13^	*U*^23^
S1	0.02002 (15)	0.01758 (16)	0.02402 (17)	0.00250 (12)	0.01091 (12)	0.00250 (12)
O1	0.0221 (4)	0.0132 (4)	0.0194 (4)	−0.0009 (3)	0.0005 (3)	−0.0023 (3)
N1	0.0165 (5)	0.0138 (5)	0.0194 (5)	−0.0013 (4)	0.0066 (4)	0.0001 (4)
N2	0.0172 (5)	0.0121 (5)	0.0197 (5)	0.0011 (4)	0.0070 (4)	0.0016 (4)
N3	0.0160 (5)	0.0194 (6)	0.0208 (5)	0.0001 (4)	0.0048 (4)	0.0000 (4)
N4	0.0191 (5)	0.0181 (6)	0.0301 (6)	0.0044 (4)	0.0119 (5)	0.0068 (5)
C1	0.0161 (6)	0.0240 (7)	0.0258 (7)	0.0004 (5)	0.0072 (5)	−0.0014 (5)
C2	0.0230 (6)	0.0245 (7)	0.0236 (6)	−0.0037 (5)	0.0114 (5)	−0.0013 (5)
C3	0.0248 (6)	0.0207 (6)	0.0180 (6)	−0.0025 (5)	0.0054 (5)	0.0018 (5)
C4	0.0179 (6)	0.0186 (6)	0.0193 (6)	−0.0007 (5)	0.0032 (4)	0.0007 (5)
C5	0.0165 (5)	0.0145 (6)	0.0171 (5)	−0.0017 (4)	0.0046 (4)	−0.0023 (4)
C6	0.0158 (5)	0.0136 (6)	0.0165 (5)	−0.0012 (4)	0.0036 (4)	−0.0016 (4)
C7	0.0158 (5)	0.0145 (6)	0.0179 (6)	0.0005 (4)	0.0032 (4)	0.0004 (5)
C8	0.0159 (5)	0.0125 (6)	0.0162 (5)	0.0012 (4)	0.0044 (4)	0.0006 (4)
C9	0.0143 (5)	0.0130 (6)	0.0163 (5)	−0.0002 (4)	0.0055 (4)	0.0007 (4)
C10	0.0152 (5)	0.0139 (6)	0.0206 (6)	0.0023 (4)	0.0033 (4)	0.0012 (5)
C11	0.0158 (5)	0.0168 (6)	0.0193 (6)	0.0013 (5)	0.0001 (4)	−0.0005 (5)
C12	0.0165 (5)	0.0141 (6)	0.0181 (6)	−0.0021 (4)	0.0059 (4)	−0.0010 (5)
C13	0.0209 (6)	0.0129 (6)	0.0204 (6)	0.0020 (5)	0.0026 (5)	0.0025 (5)
C14	0.0207 (6)	0.0161 (6)	0.0154 (5)	0.0008 (5)	0.0005 (4)	0.0022 (5)
C15	0.0161 (5)	0.0129 (6)	0.0188 (6)	−0.0004 (4)	0.0043 (4)	−0.0032 (4)
C16	0.0219 (6)	0.0227 (7)	0.0254 (7)	−0.0008 (5)	−0.0027 (5)	−0.0057 (5)
C17	0.0315 (7)	0.0262 (8)	0.0311 (8)	−0.0015 (6)	0.0010 (6)	−0.0125 (6)

### Geometric parameters (Å, º)

**Table:**

S1—C15	1.6822 (13)	C7—C8	1.5483 (16)
O1—C12	1.3726 (15)	C7—H7A	0.9900
O1—C16	1.4406 (15)	C7—H7B	0.9900
N1—C6	1.2889 (15)	C8—C9	1.5201 (17)
N1—N2	1.3906 (14)	C8—H8A	1.0000
N2—C15	1.3628 (15)	C9—C10	1.3878 (16)
N2—C8	1.4756 (15)	C9—C14	1.4032 (17)
N3—C1	1.3454 (16)	C10—C11	1.3966 (17)
N3—C5	1.3492 (16)	C10—H10A	0.9500
N4—C15	1.3341 (17)	C11—C12	1.3885 (17)
N4—H1N4	0.925 (18)	C11—H11A	0.9500
N4—H2N4	0.825 (18)	C12—C13	1.3979 (17)
C1—C2	1.3810 (19)	C13—C14	1.3799 (18)
C1—H1A	0.9500	C13—H13A	0.9500
C2—C3	1.3902 (19)	C14—H14A	0.9500
C2—H2A	0.9500	C16—C17	1.5069 (19)
C3—C4	1.3871 (17)	C16—H16A	0.9900
C3—H3A	0.9500	C16—H16B	0.9900
C4—C5	1.3964 (18)	C17—H17A	0.9800
C4—H4A	0.9500	C17—H17B	0.9800
C5—C6	1.4685 (16)	C17—H17C	0.9800
C6—C7	1.5056 (17)		
			
C12—O1—C16	117.61 (10)	N2—C8—H8A	111.0
C6—N1—N2	107.28 (10)	C9—C8—H8A	111.0
C15—N2—N1	119.74 (10)	C7—C8—H8A	111.0
C15—N2—C8	127.98 (10)	C10—C9—C14	117.94 (11)
N1—N2—C8	112.27 (9)	C10—C9—C8	123.27 (11)
C1—N3—C5	117.10 (11)	C14—C9—C8	118.69 (10)
C15—N4—H1N4	118.9 (11)	C9—C10—C11	121.48 (11)
C15—N4—H2N4	119.8 (12)	C9—C10—H10A	119.3
H1N4—N4—H2N4	121.3 (16)	C11—C10—H10A	119.3
N3—C1—C2	123.70 (12)	C12—C11—C10	119.43 (11)
N3—C1—H1A	118.2	C12—C11—H11A	120.3
C2—C1—H1A	118.2	C10—C11—H11A	120.3
C1—C2—C3	118.44 (12)	O1—C12—C11	124.56 (11)
C1—C2—H2A	120.8	O1—C12—C13	115.40 (11)
C3—C2—H2A	120.8	C11—C12—C13	120.03 (11)
C4—C3—C2	119.40 (12)	C14—C13—C12	119.62 (11)
C4—C3—H3A	120.3	C14—C13—H13A	120.2
C2—C3—H3A	120.3	C12—C13—H13A	120.2
C3—C4—C5	118.07 (12)	C13—C14—C9	121.48 (11)
C3—C4—H4A	121.0	C13—C14—H14A	119.3
C5—C4—H4A	121.0	C9—C14—H14A	119.3
N3—C5—C4	123.29 (11)	N4—C15—N2	115.59 (11)
N3—C5—C6	114.95 (11)	N4—C15—S1	124.18 (9)
C4—C5—C6	121.75 (11)	N2—C15—S1	120.24 (9)
N1—C6—C5	120.60 (11)	O1—C16—C17	107.17 (11)
N1—C6—C7	114.13 (10)	O1—C16—H16A	110.3
C5—C6—C7	125.17 (10)	C17—C16—H16A	110.3
C6—C7—C8	100.97 (9)	O1—C16—H16B	110.3
C6—C7—H7A	111.6	C17—C16—H16B	110.3
C8—C7—H7A	111.6	H16A—C16—H16B	108.5
C6—C7—H7B	111.6	C16—C17—H17A	109.5
C8—C7—H7B	111.6	C16—C17—H17B	109.5
H7A—C7—H7B	109.4	H17A—C17—H17B	109.5
N2—C8—C9	111.92 (10)	C16—C17—H17C	109.5
N2—C8—C7	100.10 (9)	H17A—C17—H17C	109.5
C9—C8—C7	111.48 (10)	H17B—C17—H17C	109.5
			
C6—N1—N2—C15	167.71 (11)	C6—C7—C8—C9	97.86 (11)
C6—N1—N2—C8	−13.34 (13)	N2—C8—C9—C10	1.69 (16)
C5—N3—C1—C2	−0.8 (2)	C7—C8—C9—C10	−109.50 (13)
N3—C1—C2—C3	0.6 (2)	N2—C8—C9—C14	178.00 (10)
C1—C2—C3—C4	−0.2 (2)	C7—C8—C9—C14	66.81 (14)
C2—C3—C4—C5	0.03 (19)	C14—C9—C10—C11	−0.20 (18)
C1—N3—C5—C4	0.56 (19)	C8—C9—C10—C11	176.13 (11)
C1—N3—C5—C6	−179.50 (11)	C9—C10—C11—C12	−0.76 (18)
C3—C4—C5—N3	−0.21 (19)	C16—O1—C12—C11	−0.39 (17)
C3—C4—C5—C6	179.86 (11)	C16—O1—C12—C13	−179.50 (11)
N2—N1—C6—C5	−179.01 (10)	C10—C11—C12—O1	−177.82 (11)
N2—N1—C6—C7	−2.46 (14)	C10—C11—C12—C13	1.26 (18)
N3—C5—C6—N1	170.77 (11)	O1—C12—C13—C14	178.37 (11)
C4—C5—C6—N1	−9.29 (18)	C11—C12—C13—C14	−0.79 (18)
N3—C5—C6—C7	−5.37 (17)	C12—C13—C14—C9	−0.19 (19)
C4—C5—C6—C7	174.57 (12)	C10—C9—C14—C13	0.68 (18)
N1—C6—C7—C8	15.72 (14)	C8—C9—C14—C13	−175.83 (11)
C5—C6—C7—C8	−167.92 (11)	N1—N2—C15—N4	1.90 (17)
C15—N2—C8—C9	82.65 (15)	C8—N2—C15—N4	−176.86 (11)
N1—N2—C8—C9	−96.19 (11)	N1—N2—C15—S1	−177.84 (8)
C15—N2—C8—C7	−159.15 (12)	C8—N2—C15—S1	3.40 (17)
N1—N2—C8—C7	22.01 (12)	C12—O1—C16—C17	177.61 (11)
C6—C7—C8—N2	−20.67 (11)		

### Hydrogen-bond geometry (Å, º)

*Cg*_1_ and *Cg*_2_ are the centroids of the C1–C5/N1 and C9–C14 rings, respectively.

**Table:**

*D*—H···*A*	*D*—H	H···*A*	*D*···*A*	*D*—H···*A*
N4—H1*N*4···S1^i^	0.925 (19)	2.472 (19)	3.3803 (12)	167.3 (15)
N4—H2*N*4···O1^ii^	0.825 (18)	2.296 (18)	3.0604 (15)	154.2 (17)
C3—H3*A*···N3^iii^	0.95	2.51	3.4053 (18)	156
C2—H2*A*···*Cg*2^iii^	0.95	2.67	3.4401 (14)	138
C7—H7*A*···*Cg*1^iv^	0.99	2.69	3.4841 (13)	138

Symmetry codes: (i) −*x*+1, −*y*+2, −*z*+2; (ii) *x*, *y*+1, *z*; (iii) −*x*+2, *y*+1/2, −*z*+3/2; (iv) −*x*+2, −*y*+2, −*z*+2.
